# Machine learning models for risk prediction of age-related macular degeneration in Fujian eye study

**DOI:** 10.1371/journal.pone.0335620

**Published:** 2025-11-04

**Authors:** Yang Li, Bin Wang, Xiangdong Luo, Mingqin Zhang, Qinrui Hu, Xiaoxin Li

**Affiliations:** 1 Eye Institute and Affiliated Xiamen Eye Center of Xiamen University, School of Medicine, Xiamen University, Xiamen, China; 2 Xiamen Clinical Research Center for Eye Diseases, Xiamen, Fujian, China; 3 Xiamen Key Laboratory of Ophthalmology, Xiamen, Fujian, China; 4 Translational Medicine Institute of Xiamen Eye Center of Xiamen University, Xiamen, Fujian, China; 5 Fujian Provincial Key Laboratory of Corneal & Ocular Surface Diseases, Xiamen, Fujian, China; 6 Xiamen Municipal Key Laboratory of Corneal & Ocular Surface Diseases, Xiamen, Fujian, China; 7 Department of Ophthalmology, Peking University People’s Hospital, Beijing, China; University of Warmia, POLAND

## Abstract

**Objective:**

Age-related macular degeneration (AMD) is a retinal disorder that significantly impairs vision. This study investigates various machine learning models for predicting AMD risk, laying the groundwork for further research using big data and determining the most effective predictive model.

**Methods:**

Utilizing data from 8211 records with 39 features from the Fujian Eye Study, a cross-sectional epidemiological investigation, several machine learning models were developed and assessed. The models included logistic regression (LR), K-nearest neighbors (KNN), support vector machine (SVM), decision tree (DT), random forest (RF), light gradient boosting machine (LightGBM), and extreme gradient boosting (XGBoost). Data preprocessing, feature selection, and model training were all key components of the study.

**Results:**

After evaluating multiple models, the logistic regression model emerged as the most accurate, achieving a balanced accuracy of 0.6364. Among the predictive features, educational background had the highest influence on the model’s predictions, with an average SHAP (SHapley Additive exPlanations) value of 0.8199. Other significant factors included outdoor time and left eye spherical equivalent (OSSE), with SHAP values of 0.6474 and 0.6377, respectively.

**Conclusion:**

This study confirms that logistic regression is the most effective machine learning model for predicting AMD risk, with educational background identified as the most critical risk factor.

## 1 Introduction

Age-related macular degeneration (AMD) is prevalent among middle-aged and elderly populations and is a leading cause of vision impairment [1]. The growing incidence of AMD, in tandem with an aging global population, poses substantial public health challenges [2]. Early detection and intervention are vital; however, traditional diagnostic methods, which rely heavily on ophthalmologists’ expertise and imaging techniques, are inherently subjective and limited. Consequently, there is a pressing need for more accurate and efficient diagnostic tools [3].

Recently, machine learning has seen extensive application and development in the medical domain [4–8]. Machine learning models offer several benefits for predicting AMD. They can autonomously process and analyze large datasets, significantly enhancing prediction efficiency while minimizing manual intervention and subjective error [3]. Furthermore, these models can achieve high-precision recognition of complex data patterns through training and optimization, improving prediction accuracy [6]. Additionally, machine learning models can offer personalized AMD risk assessments based on individual characteristics, facilitating the development of targeted prevention and treatment strategies [7]. Finally, by integrating multiple data sources, including clinical records, genetic information, and imaging data, machine learning models can provide a comprehensive risk assessment [8].

This study employed population-based epidemiological survey data to thoroughly explore AMD prediction models, aiming to identify the optimal approach. The findings not only enhance our understanding of AMD pathogenesis but also have the potential to significantly reduce the healthcare burden by providing more efficient diagnostic and treatment methods. This research is intended to serve as a reference for developing predictive models for other ophthalmic conditions and to contribute to the advancement of data-driven precision medicine in the medical industry.

## 2 Materials and methods

### 2.1 Dataset

A population based cross-sectional study was performed on 8211 residents aged 50 years and older in Fujian Province, Southeast China from May 2018 to October 2019. Random cluster sampling was used in this investigation, and the calculation formula and sample size have been reported [9]. The dataset comprised 6663 records, each with 39 features. These features encompassed basic patient information (e.g., age, gender, urbanization level (urban and rural), geographical location (coastal and inland), history of hypertension (HBP or not), hyperlipidemia (HG or not), diabetes mellitus (DM or not), educational background, occupation, outdoor activity duration, use of eye protection, income, blood type, nighttime use of mobile phones, smoking history, history of tea drinking, alcohol drinking history), ocular conditions (e.g., eye discomfort, pain history), and clinical measurements (e.g., body mass index (BMI), heart rate, systolic blood pressure (SBP), diastolic blood pressure (DBP), intraocular pressure (IOP), near visual acuity (NVA), best-corrected visual acuity (BCVA), diopters (degree of spherical mirror, S), astigmatism (cylinder, C), spherical equivalent (SE), high myopia (HM)).

### 2.2 Data preprocessing

Data preprocessing includes the following steps:

(1) Check and handle missing and infinite values:

Categorical Variables (e.g., HM, HBPornot, DMornot, education, occupation):

For all categorical variables, missing values were explicitly coded as a separate category “-1” to preserve the missingness pattern as part of the data structure. This approach avoids biased imputation for discrete variables and ensures transparency in handling missing categorical data.

Continuous Variables (e.g., HEIGHT, WEIGHT, BMI, SBP, DBP, ODIOP):

Continuous variables exhibited a low missingness proportion (<10% per feature). For these, missing values were replaced with the overall mean of the respective variable. Mean imputation was chosen because of the small proportion of missing data, minimizing distortion of the underlying distribution while retaining statistical power.

High-Missingness Variables (ODBCVA0 and OSBCVA0):

These variables had significantly higher missingness rates (>30%), rendering imputation unreliable. Consequently, rows with missing values for ODBCVA0 or OSBCVA0 were excluded from the analysis. This reduced the dataset from an initial 8,211 entries to 6,663 entries, ensuring robustness in subsequent analyses.

(2) Data standardization: All feature data was standardized to eliminate the influence of dimensional differences.

### 2.3 Feature selection

Feature importance and correlation analyses were used to identify the most impactful features for AMD prediction.

### 2.4 Model training and evaluation

We implemented seven machine learning models—logistic regression (LR), K-nearest neighbors (KNN), support vector machine (SVM), decision tree (DT), random forest (RF), LightGBM, and XGBoost—and optimized their hyperparameters using cross-validation. Model performance was rigorously evaluated through:Confusion matrix analysis, Heatmap visualization, SHAP value interpretation, Feature importance ranking.

The final dataset was randomly partitioned into training (n = 5,330) and testing (n = 1,333) sets using an 80:20 stratified sampling approach to maintain outcome distribution parity. The testing dataset comprised completely independent samples excluded from all model training and hyperparameter tuning phases. All predictions were generated exclusively on this held-out test set.

### 2.5 Ethics statement

A clinical study registry was obtained from the 2018–2019 FJES study (register number: ChiCTR2100043349, registration date: 2021-02-21) and the Human Ethics and Consent to Participate declarations was approved by the Ethics Committee of Xiamen University Xiamen Eye Center (Acceptance number: XMYKZX-KY-2018-001). All procedures were performed in accordance with the Declaration of Helsinki, and informed consent was obtained from all participants.

## 3 Results

The dataset comprised 6663 records from 8211 residents, each with 39 features.

### 3.1 Best-performing model

**Fig 1:**
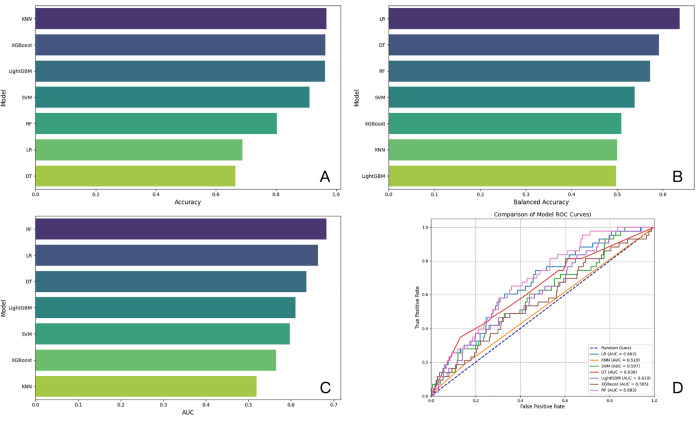
The accuracy (A), balanced accuracy (B), AUC value (C) and AUC curve (D) of seven models in this study.

Following cross-validation, the logistic regression (LR) model exhibited the highest performance, achieving the balanced accuracy of 0.6364 and F1 score of 0.1073 (see Table 1 and Fig 1).

**Table 1:** The balanced accuracy and F1 score of several models.

Model	Accuracy	Balanced accuracy	AUC	Precision	Recall	F1 score
**LR**	0.6879	0.6364	0.6630	0.0591	0.5814	0.1073
**KNN**	0.9670	0.4996	0.5192	0.0000	0.0000	0.0000
**SVM**	0.9114	0.5384	0.5972	0.0690	0.1395	0.0923
**DT**	0.6654	0.5911	0.6358	0.0492	0.5116	0.0898
**RF**	0.9167	0.5636	0.7008	0.0952	0.1860	0.1260
**LightGBM**	0.9625	0.4973	0.6104	0.0000	0.0000	0.0000
**XGBoost**	0.9640	0.5093	0.5651	0.1429	0.0233	0.0400

### 3.2 Confusion matrix

**Fig 2:**
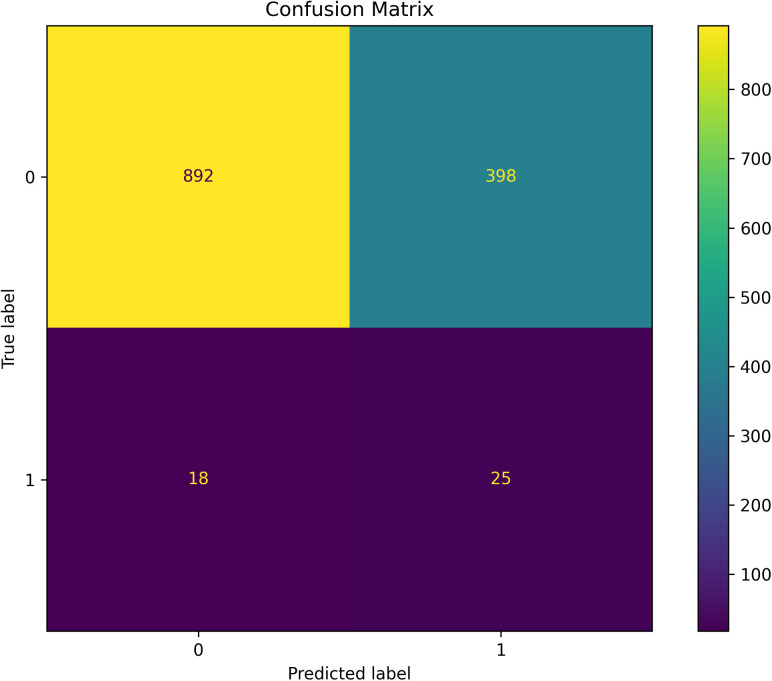
The confusion matrix analysis results of logistic regression model in this study.

To assess the classification model’s performance, a confusion matrix was generated (Fig 2). The matrix details are as follows:

- True Positive (TP), the Bottom Right Quadrant (Purple) of Fig 5: Represents the 25 samples with a true label of “1” correctly predicted as “1”.
- True Negative (TN), the Top Left Quadrant (Yellow) of Fig 5: Represents the 892 samples with a true label of “0” correctly predicted as “0”.
- False Negative (FN), the Bottom Left Quadrant (Purple) of Fig 5: Shows 18 samples with a true label of “1” incorrectly predicted as “0”.
- False Positive (FP), the Top Right Quadrant (Green) of Fig 5: Reflects 398 samples with a true label of “0” incorrectly predicted as “1”.

**Fig 3:**
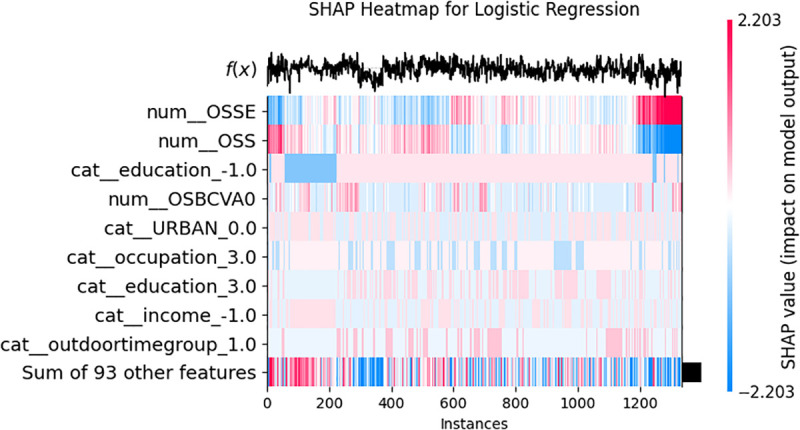
The SHAP heatmap for logistic regression model in this study.

**Fig 4:**
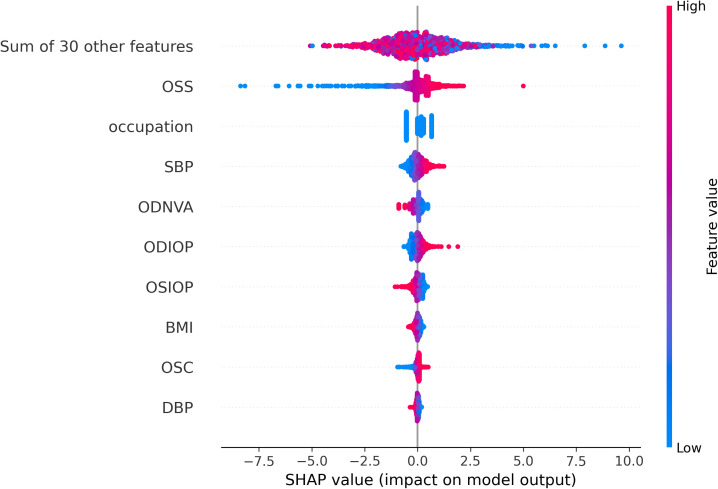
The beeswarm summary plot of impact on logistic regression model output in this study.

**Fig 5:**
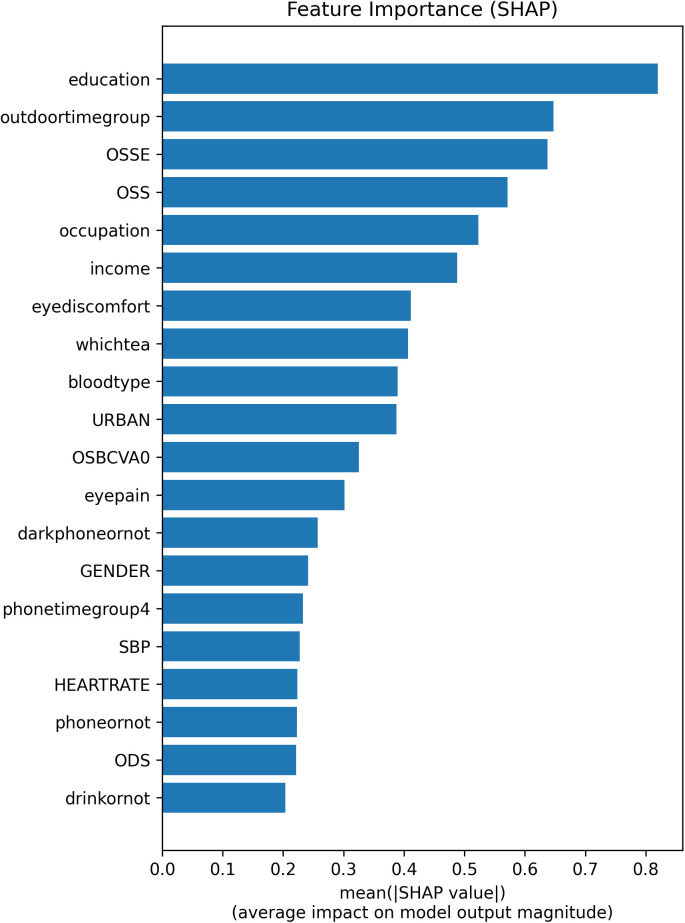
The histogram of feature importance analysis of average impact on logistic regression model output magnitude in this study.

From the confusion matrix, precision and recall rates were calculated. Precision is defined as the ratio of correctly predicted positive samples to the total predicted positive samples (TP/(TP + FP)), while recall is the ratio of correctly predicted positive samples to all actual positive samples (TP/(TP + FN)).

- Precision: 25/(25 + 398)=0.0591
- Recall: 25/(25 + 18)=0.5814
- F1 score: 2(0.0591*0.5814)/(0.0591 + 0.5814) = 0.1073

The classification model demonstrated poor prediction accuracy, as evidenced by its low precision and recall rates.

### 3.3 Heatmap analysis

In the heatmap analysis, the impact of different features on the model’s output was evaluated through color intensity (Fig 3). Typically, features with colors closer to red indicate a strong positive influence, while those nearer to blue signify a strong negative impact.

Key features identified through the heatmap include:

- Left eye spherical equivalent (OSSE): Exhibits a deep red color, indicating a substantial positive influence on the model’s predictions.
- Left Eye Sphericity (OSS): Also shows a significant negative impact, although slightly less than OSSE.
- Educational background: Depicts a considerable effect, albeit in a negative direction.

These identified features, with their respective positive or negative impacts, provide a clear understanding of the model’s predictions, which can inform further analysis and targeted interventions.

### 3.4 SHAP value analysis

The SHAP (SHapley Additive exPlanations) value analysis further explored the influence of various features on the model’s output (Fig 4). The features, listed in order of impact, include OSS, occupation, SBP, ODNVA, ODIOP (right eye IOP), OSIOP (left eye IOP), BMI, OSC, DBP and a cumulative group of 30 other features.

Key insights include:

- Positive Influence: occupation, ODNVA, OSIOP, BMI and DBP all have positive SHAP values (0.3708, 0.2014, 0.1529, 0.0.0853 and 0.0447), indicating a positive influence on the model’s predictions.
- Negative Influence: OSS, ODIOP, SBP and OSC have negative SHAP values (−0.5713, −0.1903, −0.2278 and −0.0714), highlighting a negative impact.
- Cumulative Negative Impact: The group of 30 other features collectively shows a negative SHAP value (−1.1703).

In summary, aside from the cumulative group of features, the major features have a negative SHAP value, underscoring their contribution to the model’s output. This analysis provides valuable insights that can guide future research and clinical decision-making.

### 3.5 Feature importance analysis

The feature importance analysis revealed the relative influence of different factors on the model’s predictions (Fig 5):

- Educational background: Emerged as the most influential factor, with an average SHAP value of 0.8199, considerably higher than other factors.
- outdoortime and OSSE: Both had significant impacts, with SHAP values of 0.6474 and 0.6377, respectively.
- Moderate Impact Factors: OSS, occupation, and income had moderate influences, with SHAP values around 0.5–0.6.
- Lesser Impact Factors: Features such as eye discomfort, tea type, bloodtype, urbanization, OSBCVA and eye pain had relatively lower impacts, with SHAP values ranging from 0.3 to 0.4.

The feature importance chart provides a visual representation of these impacts, with educational background identified as the most critical factor, while less impactful variables like drinking are at the lower end of the spectrum.

## 4 Discussion

### 4.1 Model performance and clinical applicability

This study confirms the utility of machine learning in predicting the risk of AMD. Among the various models evaluated, logistic regression demonstrated superior performance in predicting AMD risk, showcasing its significant potential in practical applications. This finding is consistent not only with AMD risk prediction but also with similar models in other medical domains [10–15]. For predicting AMD risk, logistic regression assigns probability values based on the weighting of features such as age, gender, blood pressure, and vision, facilitating the prediction of AMD occurrence [8,16,17]. This underscores the broader relevance of machine learning in clinical prediction and risk assessment, with future studies potentially integrating additional clinical data to enhance model generalization and accuracy. Its simplicity and clarity make it particularly useful in clinical contexts, allowing both healthcare professionals and patients to understand the underlying basis of predictions. In clinical practice, models that are easy to implement and deploy are often preferred due to their practicality. Logistic regression’s low computational cost makes it suitable for large-scale data prediction in real-time. Furthermore, its linear structure allows it to quickly adapt to new data, making it advantageous for dynamic monitoring and timely risk assessments [18].

The low population prevalence of macular degeneration (approximately 2%) poses significant challenges to the predictive accuracy of the model, as evidenced by the confusion matrix metrics (Precision = 0.059, Recall = 0.581). The high false positive rate (FP = 398) and moderate false negative rate (FN = 18) reflect the model’s limited discriminative power in imbalanced class settings. To address this, future iterations should incorporate multidimensional interactions among risk factors (e.g., education × OSSE and occupation × UV exposure identified in SHAP analyses) while integrating advanced techniques such as cost-sensitive learning or ensemble methods to enhance specificity without compromising sensitivity. This approach aligns with the need for precision public health strategies targeting rare yet impactful outcomes like AMD.

### 4.2 Risk factors

The SHAP analysis confirms educational background (SHAP = 0.8199), outdoor time (outdoor time, SHAP = 0.6474), and OSSE (SHAP = 0.6377) as the most influential risk factors for AMD, with mean absolute SHAP values exceeding 0.6. These findings align with epidemiological studies linking low education levels to limited health literacy and reduced access to preventive care, as well as prolonged UV exposure from outdoor activities accelerating retinal degeneration [19,20]. Public health strategies should prioritize addressing socioeconomic inequities (e.g., targeted education programs) and promoting UV-protective behaviors (e.g., sunglasses use) to mitigate AMD risk [21–25].

Notably, OSS (SHAP = 0.5713) and occupation (SHAP = 0.5229) rank closely behind the top three factors, suggesting occupational exposures (e.g., blue light or chemical hazards) and ocular surface stability (OSS) are critical yet understudied contributors. For instance, the interaction between num_OSSE and cat_education_1.0 in the SHAP heatmap (Fig 3) implies that individuals with lower education levels may exhibit compounded risk due to poor ocular surface health, potentially reflecting limited healthcare access or self-care practices. The isolated predictive role of left-eye spherical equivalent (SE) warrants discussion. Though interocular symmetry in refractive status is common, asymmetric AMD progression is clinically recognized due to factors like: Differential light exposure patterns (e.g., unilateral window-side seating during activities); [26,27] Unilateral cataract surgery history (affecting SE measurements and retinal light exposure); [28,29] Systemic comorbidities (e.g., carotid stenosis potentially impacting ocular perfusion asymmetrically); [30–32] Data-driven discovery: Machine learning models may detect subtle, laterally divergent SE-AMD pathophysiological relationships not yet characterized in literature.

While income (SHAP = 0.4882) and eyediscomfort (SHAP = 0.4114) demonstrate moderate effects, their clinical relevance lies in their roles as proxies for systemic health disparities and early symptomatic indicators of AMD. Conversely, features such as SBP (SHAP = 0.2278), BMI (SHAP = 0.0853), and OSC(SHAP = 0.0714) exhibit minimal individual impact, contradicting earlier hypotheses about their dominance. This discrepancy underscores the need for caution when extrapolating biological mechanisms from model outputs without clinical validation.

Lower-impact features, including phone usage habits (phonetimegroup4: SHAP = 0.2327; darkphoneornot: SHAP = 0.2570), blood type (SHAP = 0.3892), and URBAN residency (SHAP = 0.3876), collectively contribute to risk stratification, as evidenced by the aggregated effect of “Sum of 93 other features” in Fig 3. The beeswarm plot (Fig 4) further reveals bidirectional impacts: for example, OSS (SHAP = 0.5713 ↓) and ODNVA (SHAP = 0.2014 ↑) exert opposing influences on AMD risk, highlighting the multifactorial nature of disease progression.

Model performance metrics (e.g., RF AUC = 0.683, LR Balanced Accuracy = 0.6) support the reliability of these interpretations but also emphasize limitations. The moderate AUC values suggest unaccounted confounders, such as genetic predispositions or dietary factors, which may attenuate clinical applicability. Future studies should integrate multimodal data to refine risk prediction and validate SHAP-derived hypotheses through longitudinal cohorts. While the cumulative effect of these lesser factors on AMD risk might not be as strong, their inclusion in a comprehensive risk management strategy is essential for a thorough approach to the prevention and management of AMD. Understanding the complex interplay of these factors, both major and minor, will enable the development of more effective, personalized prevention and treatment strategies for individuals at risk of AMD.

### 4.3 Limitations

Firstly, the model was trained and validated exclusively on data from Fujian Province, China. While this regional focus introduces biases (e.g., genetic homogeneity, localized environmental exposures, or lifestyle patterns), it simultaneously provides unique insights into how geographically specific factors may influence AMD risk.

Secondly, the exclusion of a significant proportion of eligible records (18.85%) due to incomplete data, which may introduce selection bias and affect model generalizability. While this reflects real-world clinical data challenges, future efforts should prioritize standardized data collection protocols to minimize missing information.

Thirdly, although the current study leverages a dataset rich in important features, it primarily relies on population-based data. To enhance the accuracy of predictive models, future studies should incorporate a broader range of clinical data, such as genetic markers and hematological parameters. The integration of multimodal data will enable machine learning models to gain a more comprehensive understanding of AMD risk factors, thereby improving their generalization capabilities and robustness.

Lastly, model integration and optimization represent another promising avenue for future work. Different machine learning models excel in various data environments, and exploring techniques for integrating these models could lead to improved overall prediction performance. By combining the strengths of multiple models, future studies can achieve more accurate and reliable predictions. Moreover, the interpretability and visualization of machine learning models remain critical challenges in clinical applications. Optimizing hyperparameters and refining the training processes of these models will further enhance their accuracy and stability, ensuring that they can be effectively deployed in real-world clinical applications.

## Conclusion

This study affirms the utility of machine learning models in predicting the risk of AMD. Looking ahead, future research should focus on integrating a broader spectrum of clinical data, such as genetic and biochemical markers, with advanced machine learning algorithms. This approach will likely enhance the model’s generalizability and predictive accuracy, thereby offering stronger support for the early diagnosis and treatment of AMD. Through the ongoing refinement and optimization of these machine learning models, the broader goal of precision medicine—tailoring medical treatment to the individual characteristics of each patient—can be progressively realized. Such advancements promise to significantly improve patient outcomes and quality of life by facilitating more personalized and effective treatment strategies.

### Key messages

Previous studies have highlighted traditional statistical approaches for AMD risk factor analysis, but comparative evaluations of machine learning models for AMD prediction remain limited, particularly in diverse populations.

Logistic regression outperformed six other machine learning models in AMD risk prediction, with education level, outdoor time, and ocular parameters identified as key predictors, challenging conventional prioritization of purely clinical factors.

These findings advocate for simplified, interpretable models in clinical AMD risk stratification and underscore modifiable socioeconomic factors as actionable targets, potentially reshaping public health strategies and prompting validation in longitudinal cohorts.

## Supporting information

S1 Checklist The completed STROBE (Strengthening the Reporting of Observational Studies in Epidemiology) checklist for this manuscript. (DOCX)

S1 Data The complete dataset used for the analyses presented in this study. (CSV)

## References

[1] Fleckenstein M, Keenan TDL, Guymer RH, Chakravarthy U, Schmitz-Valckenberg S, Klaver CC, et al. Age-related macular degeneration. Nat Rev Dis Primers. 2021;7(1):31. doi: 10.1038/s41572-021-00265-2 33958600 PMC12878645

[2] Wang Y, Zhong Y, Zhang L, Wu Q, Tham Y, Rim TH, et al. Global Incidence, Progression, and Risk Factors of Age-Related Macular Degeneration and Projection of Disease Statistics in 30 Years: A Modeling Study. Gerontology. 2022;68(7):721–35. doi: 10.1159/000518822 34569526

[3] Yan Q, Weeks DE, Xin H, Swaroop A, Chew EY, Huang H, et al. Deep-learning-based Prediction of Late Age-Related Macular Degeneration Progression. Nat Mach Intell. 2020;2(2):141–50. doi: 10.1038/s42256-020-0154-9 32285025 PMC7153739

[4] Thakoor KA, Yao J, Bordbar D, Moussa O, Lin W, Sajda P, et al. A multimodal deep learning system to distinguish late stages of AMD and to compare expert vs. AI ocular biomarkers. Sci Rep. 2022;12(1). doi: 10.1038/s41598-022-06273-w PMC8850456 35173191

[5] Cheung R, Chun J, Sheidow T, Motolko M, Malvankar-Mehta MS. Diagnostic accuracy of current machine learning classifiers for age-related macular degeneration: a systematic review and meta-analysis. Eye (Lond). 2022;36(5):994–1004. doi: 10.1038/s41433-021-01540-y 33958739 PMC9046206

[6] Govindaiah A, Baten A, Smith RT, Balasubramanian S, Bhuiyan A. Optimized Prediction Models from Fundus Imaging and Genetics for Late Age-Related Macular Degeneration. J Pers Med. 2021;11(11):1127. doi: 10.3390/jpm11111127 34834479 PMC8617775

[7] Ajana S, Cougnard-Grégoire A, Colijn JM, Merle BMJ, Verzijden T, de Jong PTVM, et al, EYE-RISK Consortium. Predicting Progression to Advanced Age-Related Macular Degeneration from Clinical, Genetic, and Lifestyle Factors Using Machine Learning. Ophthalmology. 2021 Apr;128(4):587–97. doi: 10.1016/j.ophtha.2020.08.031 32890546

[8] Matsuba S, Tabuchi H, Ohsugi H, Enno H, Ishitobi N, Masumoto H, et al. Accuracy of ultra-wide-field fundus ophthalmoscopy-assisted deep learning, a machine-learning technology, for detecting age-related macular degeneration. Int Ophthalmol. 2019;39(6):1269–75. doi: 10.1007/s10792-018-0940-0 29744763

[9] Li Y, Hu Q, Li X, Hu Y, Wang B, Qin X, et al. The Fujian eye cross sectional study: objectives, design, and general characteristics. BMC Ophthalmol. 2022;22(1):112. doi: 10.1186/s12886-022-02346-6 35277140 PMC8915769

[10] Christodoulou E, Ma J, Collins GS, Steyerberg EW, Verbakel JY, Van Calster B. A systematic review shows no performance benefit of machine learning over logistic regression for clinical prediction models. J Clin Epidemiol. 2019;110:12–22. doi: 10.1016/j.jclinepi.2019.02.004 30763612

[11] Lupei MI, Li D, Ingraham NE, Baum KD, Benson B, Puskarich M, et al. A 12-hospital prospective evaluation of a clinical decision support prognostic algorithm based on logistic regression as a form of machine learning to facilitate decision making for patients with suspected COVID-19. PLoS One. 2022;17(1):e0262193. doi: 10.1371/journal.pone.0262193 34986168 PMC8730444

[12] Domínguez-Rodríguez S, Serna-Pascual M, Oletto A, Barnabas S, Zuidewind P, Dobbels E, et al, EPIICAL Consortium. Machine learning outperformed logistic regression classification even with limit sample size: A model to predict pediatric HIV mortality and clinical progression to AIDS. PLoS One. 2022;17(10):e0276116. doi: 10.1371/journal.pone.0276116 36240212 PMC9565414

[13] Shipe ME, Deppen SA, Farjah F, Grogan EL. Developing prediction models for clinical use using logistic regression: an overview. J Thorac Dis. 2019;11(Suppl 4):S574–84. doi: 10.21037/jtd.2019.01.25 31032076 PMC6465431

[14] Panda NR. A Review on Logistic Regression in Medical Research. Natl J Community Med. 2022;13(4):265–70. doi: 10.55489/njcm.134202222

[15] Lynam AL, Dennis JM, Owen KR, Oram RA, Jones AG, Shields BM, et al. Logistic regression has similar performance to optimised machine learning algorithms in a clinical setting: application to the discrimination between type 1 and type 2 diabetes in young adults. Diagn Progn Res. 2020;4:6. doi: 10.1186/s41512-020-00075-2 32607451 PMC7318367

[16] Seto H, Oyama A, Kitora S, Toki H, Yamamoto R, Kotoku J, Haga A, Shinzawa M, Yamakawa M, Fukui S, Moriyama T. Gradient boosting decision tree becomes more reliable than logistic regression in predicting probability for diabetes with big data. Sci Rep. 2022 Oct 11;12(1):15889. doi: 10.1038/s41598-022-20149-z. Erratum in: Sci Rep. 2022 Dec 30;12(1):22599. doi: 10.1038/s41598-022-27052-7. 36220875 PMC9553945

[17] Schmidt-Erfurth U, Bogunovic H, Sadeghipour A, Schlegl T, Langs G, Gerendas BS, et al. Machine Learning to Analyze the Prognostic Value of Current Imaging Biomarkers in Neovascular Age-Related Macular Degeneration. Ophthalmol Retina. 2018;2(1):24–30. doi: 10.1016/j.oret.2017.03.015 31047298

[18] Chen Y, Zhu Z, Cheng W, Bulloch G, Chen Y, Liao H, et al. Choriocapillaris Flow Deficit as a Biomarker for Diabetic Retinopathy and Diabetic Macular Edema: 3-Year Longitudinal Cohort. Am J Ophthalmol. 2023;248:76–86. doi: 10.1016/j.ajo.2022.11.018 36436548

[19] Kuan V, Warwick A, Hingorani A, Tufail A, Cipriani V, Burgess S, et al, International AMD Genomics Consortium (IAMDGC). Association of Smoking, Alcohol Consumption, Blood Pressure, Body Mass Index, and Glycemic Risk Factors With Age-Related Macular Degeneration: A Mendelian Randomization Study. JAMA Ophthalmol. 2021;139(12):1299–306. doi: 10.1001/jamaophthalmol.2021.4601 34734970 PMC8569599

[20] Mao F, Yang X, Yang K, Cao X, Cao K, Hao J, et al. Six-Year Incidence and Risk Factors for Age-Related Macular Degeneration in a Rural Chinese Population: The Handan Eye Study. Invest Ophthalmol Vis Sci. 2019;60(15):4966–71. doi: 10.1167/iovs.19-27325 31790559

[21] Heesterbeek TJ, Lorés-Motta L, Hoyng CB, Lechanteur YTE, den Hollander AI. Risk factors for progression of age-related macular degeneration. Ophthalmic Physiol Opt. 2020;40(2):140–70. doi: 10.1111/opo.12675 32100327 PMC7155063

[22] Pugazhendhi A, Hubbell M, Jairam P, Ambati B. Neovascular Macular Degeneration: A Review of Etiology, Risk Factors, and Recent Advances in Research and Therapy. Int J Mol Sci. 2021;22(3):1170. doi: 10.3390/ijms22031170 33504013 PMC7866170

[23] Lee J, Kim U-J, Lee Y, Han E, Ham S, Lee W, et al. Sunlight exposure and eye disorders in an economically active population: data from the KNHANES 2008-2012. Ann Occup Environ Med. 2021;33:e24. doi: 10.35371/aoem.2021.33.e24 34754485 PMC8367748

[24] He J, Liu Y, Zhang A, Liu Q, Yang X, Sun N, et al. Joint effects of meteorological factors and PM2.5 on age-related macular degeneration: a national cross-sectional study in China. Environ Health Prev Med. 2023;28:3. doi: 10.1265/ehpm.22-00237 36631073 PMC9845061

[25] Deng Y, Qiao L, Du M, Qu C, Wan L, Li J, et al. Age-related macular degeneration: Epidemiology, genetics, pathophysiology, diagnosis, and targeted therapy. Genes Dis. 2021;9(1):62–79. doi: 10.1016/j.gendis.2021.02.009 35005108 PMC8720701

[26] Simons K. Artificial light and early-life exposure in age-related macular degeneration and in cataractogenic phototoxicity. Arch Ophthalmol. 1993;111(3):297–8. doi: 10.1001/archopht.1993.01090030015002 8447727

[27] Villegas-Pérez M. Exposición a la luz, lipofuschina y degeneración macular asociada a la edad. Arch Soc Esp Oftalmol. 2005;80(10). doi: 10.4321/s0365-66912005001000002 16245192

[28] Bhandari S, Chew EY. Cataract surgery and the risk of progression of macular degeneration. Curr Opin Ophthalmol. 2023;34(1):27–31. doi: 10.1097/ICU.0000000000000909 36484207 PMC9752199

[29] Yang L, Li H, Zhao X, Pan Y. Association between Cataract Surgery and Age-Related Macular Degeneration: A Systematic Review and Meta-Analysis. J Ophthalmol. 2022;2022:6780901. doi: 10.1155/2022/6780901 35573811 PMC9098349

[30] Smith RT, Olsen TW, Chong V, Kim J, Hammer M, Lema G, et al. Subretinal drusenoid deposits, age-related macular degeneration, and cardiovascular disease. Asia Pac J Ophthalmol (Phila). 2024;13(1):100036. doi: 10.1016/j.apjo.2024.100036 38244930

[31] Mordechaev E, Jo JJ, Mordechaev S, Govindaiah A, Fei Y, Tai K, et al. Internal Carotid Artery Stenosis and Ipsilateral Subretinal Drusenoid Deposits. Invest Ophthalmol Vis Sci. 2024;65(2):37. doi: 10.1167/iovs.65.2.37 38407857 PMC10902875

[32] Diprose WK, Wang MTM, Reidy J, Ma A, Brodie J, Steinfort B. Ophthalmic artery stenosis on three-dimensional rotational angiography: Interrater agreement, prevalence, and risk factors. Interv Neuroradiol. 2024;15910199241233020. doi: 10.1177/15910199241233020 38387875 PMC11571139

